# Interaction between Flow Diverter and Parent Artery of Intracranial Aneurysm: A Computational Study

**DOI:** 10.1155/2017/3751202

**Published:** 2017-10-25

**Authors:** Wenyu Fu, Qixiao Xia

**Affiliations:** ^1^College of Mechanical and Electrical Engineering, Beijing Union University, Beijing 100020, China; ^2^Beijing Engineering Research Center of Smart Mechanical Innovation Design Service, Beijing 100020, China

## Abstract

To evaluate the influence of deployment strategy on the mechanical interaction between braided stent and parent artery of intracranial aneurysm (the elasticity of the arterial wall is considered), finite-element analyses are carried out by referring to computational models of flow-diverter device and arterial wall. Two implantation strategies are used to virtually implant the braided stent into the ideal intracranial aneurysm model. One is the noncompacted implantation method, and the other is the implantation method of using push-pull technique. During the process of the implantation, the changes of the arterial shape around the aneurysm and the changes of the wall pressure at the contact area between the braided stent and the inner wall of the artery are analyzed. The results indicate that the average contact pressure in the area of low porosity is 57 mmHg using the push-pull technique, and the average contact pressure of the parent artery is 10.45 mmHg using the non-push-pull technique. The diameter of the parent artery at the aneurismal orifice increased about 0.2 mm when using the push-pull technique, so the elasticity of the vessel should be considered in the mechanical analysis of interaction between stent and vessel.

## 1. Introduction

An intracranial aneurysm is a pathological dilatation of the cerebral artery wall. This pathology is characterized by the unilateral or circumferential dilatation of the vessel lumen. Wide-necked or fusiform cerebral aneurysm can be effectively treated by endovascular intervention of using flow diverter [1–4] which is self-expandable, densely braided metallic stents. Braided stents with low porosity placed in the orifice of an aneurysm act as a physical barrier which can effectively reduce blood flow velocity in the aneurismal cavity, promote thrombosis formation, and achieve the treatment of cerebral aneurysm.

Researchers use geometrical projection method for virtual stent deployment into intracranial arteries [5–8]. Geometrical projection method is time-saving and easy to use, making it easy for clinicians to judge the treatment outcome quickly. However, this method does not take into account the interaction between the stent and the vessel and the shape change of the stent and vessel caused by this interaction. Previous studies [9, 10] have proven that the geometry of the stent in its deployed state plays a major role in the way the blood flow in the aneurismal cavity is diverted. Ma et al. use finite element method (FEM) to deploy braided stent into intracranial parent artery [11, 12]. Their method simulates the entire process of diverter device into parent artery and takes into account the interaction between the stent and the vessel. However, vessel wall is assumed as rigid in their study. Thus, it is impossible to obtain the local shape change of vessel (around aneurysm) during and after the implantation of the braided stent. Bock et al. use FEM to study the influence of stent structure and vessel geometry for stent-assisted coiling of intracranial aneurysms [13]. Two generic closed-cell stents and an open cell Neuroform stent are used in their study, and the porosity of the aneurysm neck is high (from 88.2% to 93.5%). The material properties of the vessel wall and stent are considered and the change of vessel geometry is obtained in their study. Conti performs numerical simulations between the wire stent and the vessel (no stenosis and no aneurysm). Using the steps of deploying stent which is different from clinical practice, numerical calculation of the interaction between the braided stent and the vessel is carried out. The shape of the deformed vessel and the stress of the vessel wall under the interaction between the deployed stent and the vessel are obtained [14]. Dottori et al. use finite element approach to investigate the interaction between the braided self-expandable Carotid Wallstent and the vessel (different geometries of the stenotic carotid segment). Their results indicate that radial force exerted by the braided self-expandable stent in the deployment phase on the vessel wall may be not sufficient for inducing a satisfactory reduction of the stenosis degree. To further reduce the stenosis degree when a braided self-expandable stent is employed, a postdilatation angioplasty should be performed [15]. Zhao et al. study the braided and the welded wire stents which are crimped and deployed into a stenosed artery using finite element analysis. They found that the radial strength of the braided stent is less than the welded one. Therefore, the deployed braided stent is more conformed to the anatomic shape of the lesion and much less efficient for restoring the patency of the stenotic artery [16]. After a comprehensive review of the existing literature, there are few papers about the interaction between the braided stent of the flow diverter and the intracranial aneurysm.

In this paper, FEM is employed to investigate the interaction between the braided stent of the flow diverter and the intracranial aneurysm. The high-fidelity virtual stenting method (HiFiVS) [12] which is based on finite element analysis is used to virtually deploy braided stent in an ideal aneurismal model in this study. One deployment strategy is noncompacting with HiFiVS and the other is compacting with HiFiVS. In the previous studies of virtual deployment braided stent using HiFiVS method [11, 12, 17], the vessel wall is treated as rigid. For comparison, FEM calculation wherein vessel wall is assumed as rigid in this study is also carried out to investigate the interaction between the braided stent of the flow diverter and the intracranial aneurysm.

Attentions are paid to the effect of the interaction between the braided stent and the intracranial aneurysm on the local shape change of the vessel, the wall pressure at the contact area between the braided stent and the inner wall of the artery, and the Von Mises stress of the stent during the process of the deployment of the braided stent. Results of the present study may help understand the treatment mechanism for the intracranial aneurysm using braided stent and its clinical practice for the selection of deployment strategy.

## 2. Materials and Methods

### 2.1. Aneurismal Model

**Figure 1:**
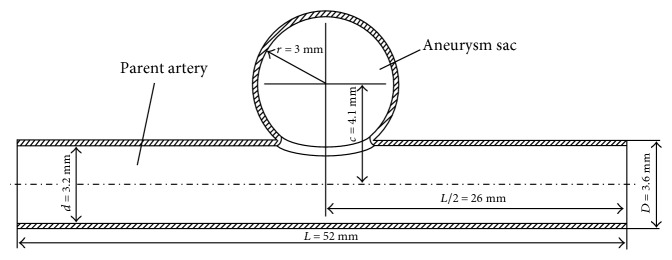
3D model of ideal intracranial aneurysm.

An ideal cerebral aneurismal model is created using SolidWorks 2016 CAD software (SolidWorks Corp., Concord, MA). A schematic representation of aneurysm geometry and relevant dimensions are shown in Figure 1. A straight pipe is used to represent the parent artery and a partial sphere to represent the aneurysm sac. The inner diameter of the parent artery is 3.2 mm, and its length is 52 mm. The inner radius of the spherical aneurysm in this study is 3 mm and the width at the aneurismal orifice is 2.52 mm. The vessel wall thickness is 0.2 mm. A sidewall aneurysm model with aforementioned dimensions is chosen because this type of aneurysm is very common in patients. The parent artery is modeled by solid element, and the element type is reduced integration eight-node linear brick elements (C3D8R). The total elements for the parent artery are 128,382. For aneurismal model, material properties are assumed to be homogeneous and described by an almost incompressible linearly elastic isotropic behavior. Its density is 1120 kg/m^3^, Young's modulus is 1.74 MPa, and Poisson's ratio is 0.499 (approximate to an incompressible material). Axial displacement at both ends of the vessel wall surface is set to 0.

### 2.2. Braided Stent Model

**Figure 2:**
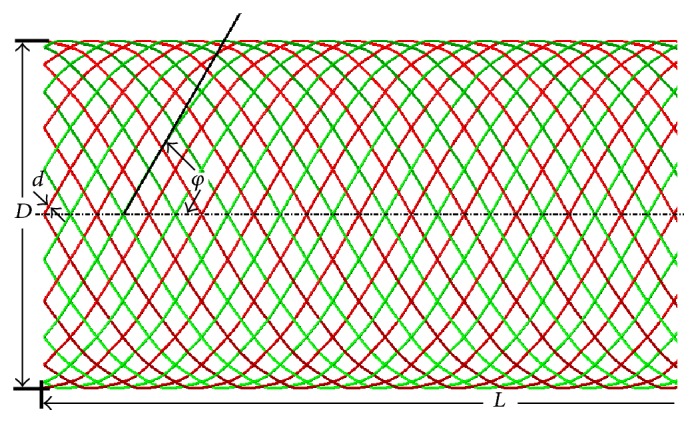
3D model of braided wire stent (*D*, nominal diameter of stent; *d*, strand diameter; *L*, nominal stent axial length; *φ*, braiding angle).

**Figure 3:**
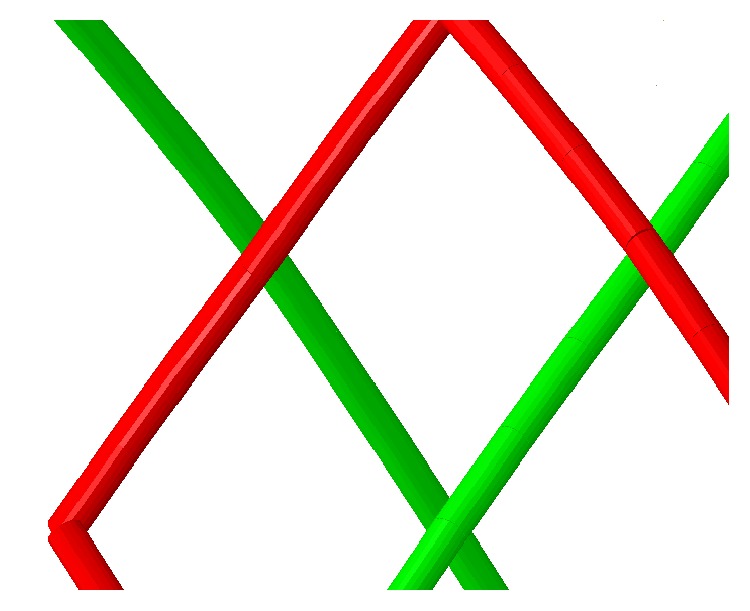
Contact model of braided wire (red, left-handed helical strands; green, right-handed helical strands).

Commercial finite element method (FEM) code ABAQUS/Explicit 6.14 (SIMULIA, Providence, RI) is used to create the geometrical and finite element model of the braided stent. The method of creating braided stent mainly includes two steps. First, the flat weaved wire stents are created. Second, the flat weaved stents are wrapped into cylindrical shape using ABAQUS plug-in wrap mesh. The model of the braided stent is shown in Figure 2. It is braided with 48 cobalt-chromium-nickel (Co-Cr-Ni) alloy strands in a helical form with a braiding angle of 75°. The wire diameter is 0.026 mm which is similar to the pipeline embolization device (Covidien, Irvine, CA). The nominal dimension of the stent is 3.5 mm in diameter and 14 mm in length. The stent is meshed with B31 beam element, which allows for large axial strains as well as transverse shear deformation. In order to reduce the dependence of the calculation results on the number of meshes, 4 simulations with different numbers of meshes are carried out. When the relative error of the Von Mises stress at the same location for two consecutive mesh models is less than 5%, it is considered that the independence of the grid has been reached. Based on the grid sensitivity analyses and considering the computational cost, a mesh size of 0.08 mm is adopted for the braided stent. The total mesh number of the stent model is 21,312. The contact model of crossing beams is depicted in Figure 3.

Co-Cr-Ni material properties obtained from the literature [11] are used to all stent's wires. Mechanical parameters are presented as follows: Young's modulus 20,600 MPa, density 8000 kg/m^3^, 0.2% yield strength 2800 MPa, isotropic hardening slope 8800 MPa, and Poisson's ratio 0.26.

### 2.3. Virtual Stent Deployment Procedure

The HiFiVS method is used to virtually deploy the braided stents in this study [11, 12]. First, unloaded braided stent is crimped from its natural nominal diameter (e.g., 3.5 mm) to a smaller size (e.g., 0.5 mm). Then, the crimped braided stent is virtually fitted into the microcatheter and let it expand to the inner surface of the microcatheter [11]. Then, the assembly of stent-microcatheter is inserted to the aneurismal model, and the stent is released from the microcatheter. The interaction property between the stent and the artery is specified as penalty (Coulomb friction) and the frictional coefficient is assumed to be 0.08. The contact algorithms used for the wire are general contact, and the corresponding algorithms for the stent and artery are contact pair. The detailed information can be found in the method section in the reference [11]. The simulation of the deployment process is quasistatic. To ensure a quasistatic simulation, the kinetic energy of the stent should not exceed 5–10% of its internal energy throughout most of the process. To achieve this, a smooth step amplitude curve is used in the numerical calculation and appropriate step time is adopted after several calculations. When the simulation is completed, the ratio between the kinetic energy and the internal energy for the stent is calculated and found to be less than 0.05, thus confirming the quasistatic nature of the simulation.

**Figure 4:**
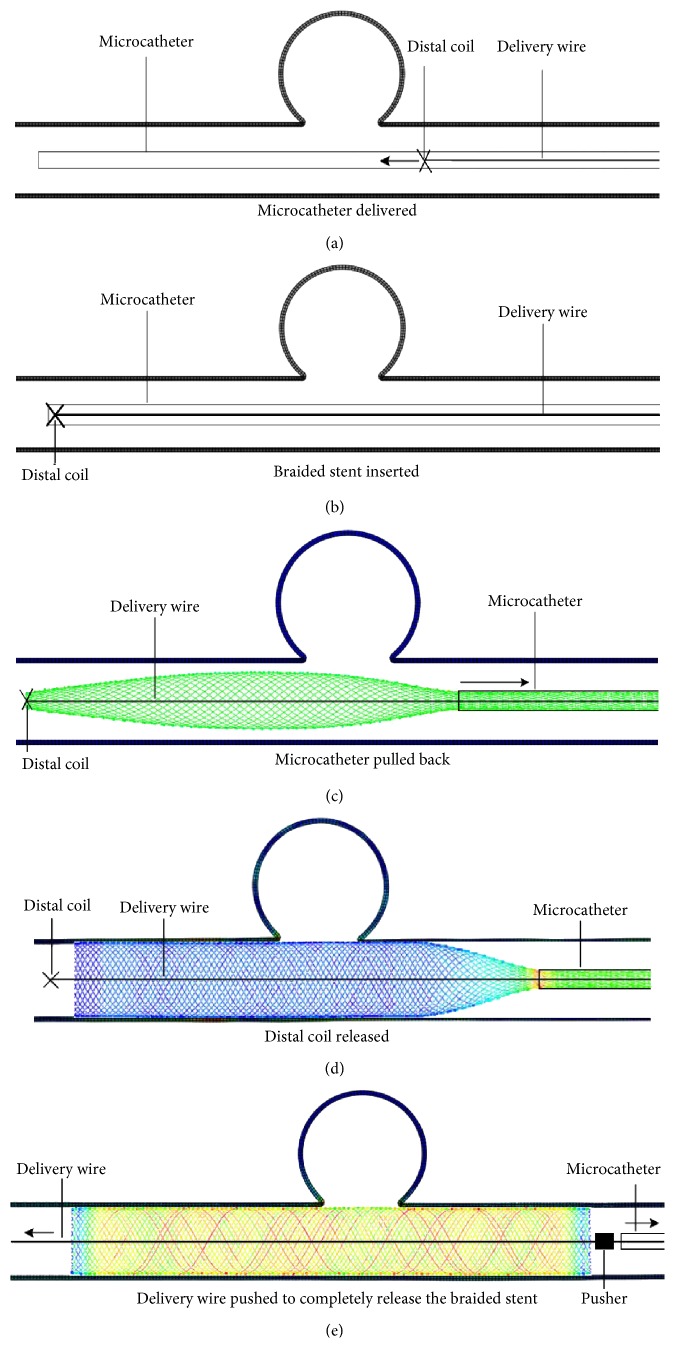
Deployment procedure of flow diverter without compaction.

**Figure 5:**
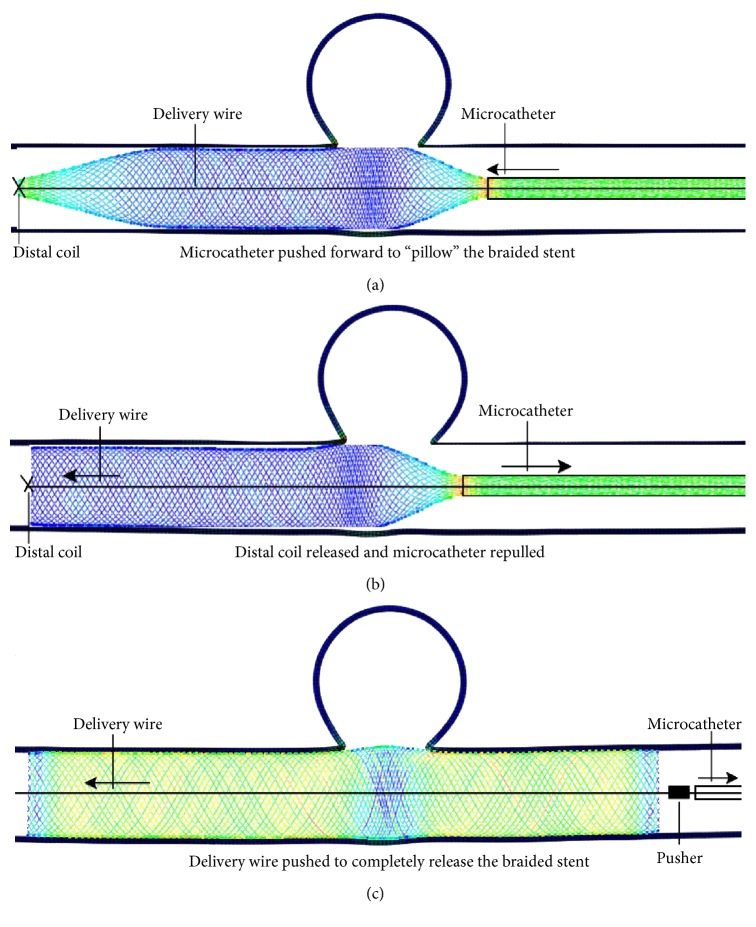
Deployment procedure of flow diverter with compaction.

Two strategies of virtual deployment are used. One is implantation without compaction and the other is implantation with compaction. Compaction is defined as follows: after the assembly, the stent-microcatheter is delivered to a proper location, the microcatheter is pulled back while the distal end of the stent is fixed by the distal coil (as shown in Figure 4(c)). Then, the microcatheter has gone forward while the stent's distal end is still fixed. The partial released stent (metal wires) is made to compact each other and stent filaments become dense across the aneurysm orifice (as shown in Figure 5(a)). This phenomenon is most pronounced in the portion of the stent near the distal end of the microcatheter. The delivery wire is not explicitly modeled in the numerical calculation and it is represented by the pathway (the center line of the parent artery) that the deployed braided stent should be passed through. A reference point is used to mimic the distal coil and it can constrain the motion of the stent's distal end. As mentioned earlier, it can fix the distal end of the stent when the microcatheter is moved. A cylinder with rigid element mesh is created as the pusher at the proximal end of the stent (as shown in Figures 4(e) and 5(c)). When the microcatheter retracts, the pusher can prevent the stent from moving along with the microcatheter. The detailed process of deployment is shown in Figures 4 and 5. The first three steps of deployment with compaction are the same as those shown in Figure 4. So they are omitted in Figure 5.

## 3. Results

Porosity, the Von Mises stress of the stent, the wall pressure, and the diameter of the vessel in the orifice of the aneurysm are compared and analyzed during the process of the implantation (two different deployment procedures).

As shown in Figures 4(e) and 5(c), the porosity is different. In the constrained state after virtual stent deployment, the porosity of the braided stent is 72.7% (without compaction) and 53.2% (with compaction), respectively. Substract ratio of the area of the metal wire (covering the aneurismal orifice) to the area of the aneurismal orifice after the stent is deployed from one and the porosity is obtained. The calculation method of the porosity is as follows: after the stent is deployed, a picture containing the stent and aneurysm is taken (adjust the model position so that the largest area of the aneurismal orifice is displayed on the computer screen). Then, the number of pixels in the aneurismal orifice is counted which is occupied by the stent and the total number of pixels is also counted in the aneurismal orifice. The value of one minus the ratio of these two numbers is the porosity. In this way, the porosity under two different implantation strategies (without compaction and compaction) is obtained.

**Figure 6:**
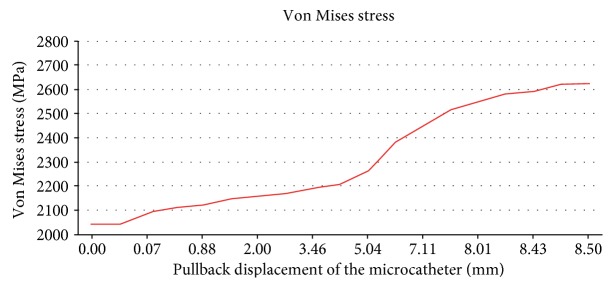
Von Mises stress of the stent (with compaction).

**Figure 7:**
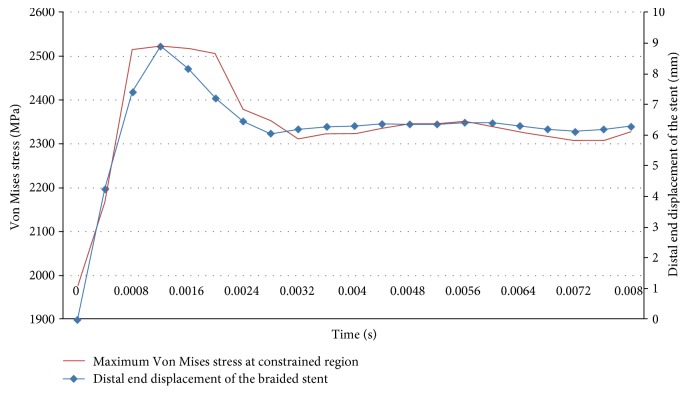
Von Mises stress and distal end displacement of the stent (without compaction).

As shown in Figures 4(d), 5(a), and 5(b), the maximum value of the Von Mises stress of the stent occurs at the boundaries of the region where the stent releases from the microcatheter and the region where the microcatheter constrains the stent during the process of the stent implantation. For the implantation approach with compaction, the maximum Von Mises stress of the braided stent occurs at the stage where the microcatheter is pulled back. The maximum Von Mises stress of the braided stent is shown in Figure 6(). For the implantation approach without compaction, the maximum Von Mises stress of the braided stent occurs at the stage where the distal coil is released. As shown in Figure 7(), the maximum Von Mises stress of the braided stent (at the border region where the constrained stent is just released from the microcatheter) increases from 1975 MPa to the 2525 MPa in the early stage of distal coil releasing (from 0 s to the 0.0012 s, the whole time of releasing distal coil is 0.008 s). At the same time, the axial length of the stent is decreased by 8.9 mm as the diameter of the stent at the distal end increases from small to the inner diameter of the vessel (3.2 mm). Subsequently, the maximum Von Mises stress decreases from 2525 MPa to 2353 MPa (from 0.0012 s to the 0.0028 s), and finally, this value tends to stabilize (2300 MPa or so).

**Figure 8:**
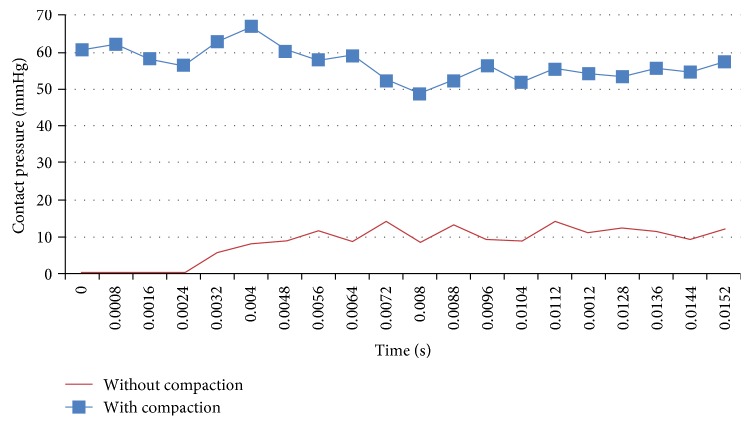
Mean contact pressure of the vascular wall in the aneurismal orifice.

During the release of the stent from the microcatheter, it will gradually contact with the inner wall of the parent vessel. So this interaction between the stent and the vessel will produce contact pressure which is exerted to the inner wall of the vessel. After analyzing the results of the calculations, it is found that the contact pressure at the aneurismal orifice in the last step (release the stent from the microcatheter) is the highest. Therefore, the contact pressure of the parent artery at the aneurismal orifice is statistically analyzed and the mean value is calculated. As shown in Figure 8(), the average value of contact pressure is 10.45 mmHg in the deployment procedure without compaction. The corresponding value is 57 mmHg in the deployment procedure with compaction.

Although the elasticity of the vessel wall is considered in the two deployment procedures, deformation size of the vascular wall near the aneurismal orifice is different. As shown in Figure 4(e), the deformation size is almost zero in the deployment procedure without compaction. As shown in Figure 5(c), the deformation in the vessel wall in the middle of the aneurismal orifice is evident (increases 0.2 mm in diameter) in the deployment procedure with compaction.

## 4. Discussions

For the HiFiVS method with compaction, of course, a lower porosity can be obtained in the orifice of an aneurysm (less than 70%). The longer the length of the microcatheter is pulled back, the lower the porosity near aneurismal orifice is. However, the Von Mises stress of the stent is increased as the length of the microcatheter which is pulled back increases as shown in Figure 6. When the length of the microcatheter which is pulled back is 8.5 mm, the maximum Von Mises stress of the stent is more than 2600 MPa. If the length of the microcatheter which is pulled back increases more than 8.5 mm, the maximum Von Mises stress of the stent will increase rapidly and exceed the yield limit of the material. Thus, the stent will enter the plastic deformation stage and its geometrical structure will be unstable. If the stent loses its stable structure, it cannot be used to treat aneurysm. In this study, the length of the microcatheter which is pulled back is 8.5 mm (the maximum value), and the corresponding minimum porosity is 53.2%. If the length of the microcatheter which is pulled back is less than 8.5 mm, the corresponding porosity will be greater than 53.2% and less than 72.7% (without compaction). Therefore, the length of the microcatheter which is pulled back must be appropriate. It is necessary to ensure that a lower porosity can be obtained at the aneurysm orifice, and at the same time, the stress of the stent cannot exceed the yield limit of the material.

It has been noted that a braided stent with a porosity of 70% significantly affects the hemodynamics in the aneurysm cavity [18]. A braided stent with a 65% actual porosity at the aneurismal orifice can predict 95% of angiographic aneurysm occlusions in rabbits [19]. In this study, porosity at aneurismal orifice is different when using two different deployment procedures. One is 72.7% (without compaction) and the other is 53.2% (with compaction). Therefore, probability using the braided stent with compaction deployment procedure for stasis and thrombosis of intra-aneurysm is more likely.

As shown in Figure 8, the contact pressure of the parent artery in the orifice is very different because of the two different implantation methods. One is 57 mmHg (with compaction), and the other is 10.45 mmHg (without compaction). The interaction between the stent and the vessel can cause vascular injury, which may lead to intimal hyperplasia in the parent artery. It is the main reason for the stenosis of the stent after bare stent is implanted. Two different implantation methods result in a large difference in contact pressure (close to 5.5 times). The contact pressure will always be present after the implantation due to self-expanding property of the braided stent. This will inevitably lead to different vascular injury of the parent artery. Damiano et al. point out that compacting a single flow diverter could be a superior strategy to overlapping multiple flow diverters, from both a biologic and economic standpoint [17]. Overlapping multiple metal devices increases the risk for in-stent thrombosis and stenosis [17, 20]. However, from the results of this study, compacting a single flow diverter will lead to greater contact pressure of the parent artery at the aneurismal orifice. It may also increase the risk for in-stent thrombosis or stenosis. There is no medium- and long-term clinical data concerning this problem. More attention should be paid to obtain more clinical data about this problem.

In this study, it is assumed that the material of the vessel wall is elastic rather than rigid. The vessel wall is also assumed as a rigid material in study process for comparison. It is found that the force and the deformation of the stent and the vessel wall during the interaction between the vessel wall and the released stent are little different in the implantation without the compaction. But there are two different points in the implantation with the compaction of the microcatheter. The first is the maximum length of the microcatheter which can be pulled back is small when the vessel wall is treated as rigid (comparison with the assumption that vessel wall is treated as elastic). For example, the maximum length of the microcatheter which can be pulled back is 8.5 mm (the vessel wall is assumed as elastic). The corresponding value is 7 mm (the vessel wall is treated as rigid). The length of the microcatheter which can be pulled back is different, and the porosity of the stent at aneurismal orifice is different. Porosity is different, and the characteristics of the blood flow inside aneurysm will be different. Why is the length of the microcatheter which can be pulled back longer (the vessel wall is assumed as elastic)? It could be that the elastic wall can absorb a certain amount of energy which is released by the crimped stent and the maximum stress of crimped stent is reduced at the position where the stent is released from the microcatheter. The second is that the diameter of parent artery at aneurismal orifice increases about 0.2 mm when the implantation of the stent is completed (the vessel wall is treated as elastic). Although the change in diameter is small, the diameter of parent artery after deformation is different. This change will affect the characteristics of blood flow in the aneurismal cavity. Therefore, the elasticity of the blood vessel should be considered when implanting the braided stent using the method with the compaction.

## 5. Limitation

It should be noted that the numerical calculation of the stent implantation process does not take into account the interactions between the blood flow and the stent or vessel wall. As the difference in diameter between the wire of the stent and the vessel is more than two orders of magnitude, it is very difficult to perform coupling calculation between the blood flow and the vessel wall or stent using the existing computing technology. As noted in the reference [12], it is the geometrical constraints and the displacement boundary condition that enforce the deployed braided stent shape regardless of the additional drag force caused by the blood flow. Since the cross-sectional area of the braided stent is small (the radius of the braided metal wire is 0.013 mm), the drag force caused by the blood flow is very small. Therefore, the drag effect on the deployed braided stent geometry is negligible.

Although the arteries are a layered anisotropic structure, the arterial tissue has been modeled as a linearly elastic isotropic material, without considering nonlinear anisotropic response induced by the tissue microstructure. Valencia et al. note that the calculation with linear elastic material overestimates the displacement by 7% with nonlinear material model [21]. In this study, the radial maximum deformation of the vessel due to the interaction between the stent and the arterial vessel is 0.2 mm. So the difference in the size of the vascular deformation caused by using a linearly elastic isotropic material is small. For Von Mises stress, when the arterial deformation increases (strain exceeds 10%), the nonlinear effect of the material is very significant [21]. In this study, the maximal arterial strain is less than 10% (0.2/3.2 = 0.0625) when implanting the braided stent using the method with the compaction. So linearly elastic isotropic material model is used for the arterial vessel in this study.

In addition, the effect of residual stress/strain on the interaction between the stent and the vessel is not considered. The braided stent is elastically deformed during the process of the crimping, and therefore, no residual stresses are generated. Residual stresses will arise from the process of stent manufacturing. But the residual stress can be greatly reduced by tempering and stability treatment after wire drawing [22]. So residual stress is not considered in this study. It should be noted that there is no direct validation about the interaction calculation between the stent and the parent artery. But the HiFiVS method used in this study has been validated as described in the reference [12]. This lays a solid foundation for this study.

## 6. Conclusions

The results of this study indicate that the arterial wall will suffer greater contact pressure in the area where the porosity is small using the implantation strategy with pulling back microcatheter. This situation may lead to in-stent thrombosis or stenosis in the area where the porosity is small. Using the implantation strategy with pulling back microcatheter, the maximum stress suffered by the stent grows rapidly with the increase of the displacement of the microcatheter which is pulled back. Therefore, the displacement that the microcatheter is pulled back must have a threshold value in clinical practice; otherwise, the stent will enter the plastic stage and the stent may lose its stability of structure. In addition, the elasticity of the vessel should be considered in the mechanical analysis of the interaction between the stent and the vessel using the implantation strategy with pulling back microcatheter. The deformation of the vessel cannot be neglected in the area where the porosity is small, which will affect blood flow in aneurismal cavity.
